# Knowledge, attitude, and practices on COVID-19 prevention and diagnosis among medical workers in the radiology department: A multicenter cross-sectional study in China

**DOI:** 10.3389/fpubh.2023.1110893

**Published:** 2023-03-02

**Authors:** Qiyuan Sun, Chunyan Yu, Zhedong Zheng, Qiong Wu, Jian Zhang, Peng Jiang, Ying Liu

**Affiliations:** ^1^Department of Medical Imaging, Longgang District Central Hospital of Shenzhen, Shenzhen, China; ^2^Department of Radiology, Shenzhen People's Hospital, Shenzhen, China; ^3^Department of Radiology, The Affiliated Hospital of BeiHua University, Jilin, China; ^4^Department of Medical Imaging, Guilin People's Hospital, Guilin, China; ^5^Department of Medical Imaging, Chinese and Mongolian Hospital of Zhalantun, Zhalantun, China

**Keywords:** COVID-19, medical staff, knowledge, attitude, practice, pandemic reaction, preventive measures, cross-sectional study

## Abstract

**Objective:**

The aim of this study was to investigate the knowledge, attitude, and practice (KAP) of medical workers in the radiology department toward the prevention and diagnosis of COVID-19.

**Methods:**

This multicenter cross-sectional study was conducted among medical workers in the radiology department of 17 hospitals between March and June 2022.

**Results:**

A total of 324 medical workers were enrolled. The mean knowledge scores were 15.3 ± 3.4 (out of 23), attitude scores were 31.1 ± 5.6 (range 8–40), and practice scores were 35.1 ± 4.4 (range 8–40). Positive attitudes (OR = 1.235, 95% CI: 1.162–1.311, *P* < 0.001) and aged 41–50 years were independently associated with higher practice scores. Those with the better practice were more likely to be older (OR = 2.603, 95% CI: 1.242–5.452, *P* = 0.011), nurses (OR = 2.274, 95% CI: 1.210–4.272, *P* = 0.011) and with junior/intermediary/vice-senior title (OR = 2.326, 95% CI: 1.030–5.255, *P* = 0.042; OR = 2.847, 95% CI: 1.226–6.606, *P* = 0.015; OR = 4.547, 95% CI: 1.806–11.452, *P* = 0.001, respectively). Subgroup analysis revealed significant differences in knowledge between technicians and physicians and nurses and between staff working in tertiary hospitals and non-tertiary hospitals. Knowledge is positively correlated with attitude (β = 0.54, *P* < 0.001), and attitude is positively correlated with practice (β = 0.37, *P* < 0.001). Attitudes significantly mediated the association between knowledge and practice (β = 0.119, *P* < 0.001).

**Conclusion:**

The radiology medical workers showed moderate knowledge but good attitudes and practices of prevention and diagnosis of COVID-19. Attitudes were found to be positively associated with better practices of prevention and diagnosis of COVID-19. Attitudes significantly mediated the association between knowledge and practice.

## Introduction

Healthcare workers are on the front lines and are particularly vulnerable to SARS-CoV-2 infection (1–3). The highly infectious SARS-CoV-2 virus poses an additional hazard to the healthcare system in addition to the burden of extended work hours, physical and psychological stress, burnout, and fatigue (4–6). In China, medical resources were once strained because of the large number of cases and the construction of temporary COVID-19-dedicated hospitals (7, 8), but early and strict measures and quick responses in China limited the infections among medical workers (9). Indeed, the protection of medical workers from occupational exposure is a key part of the epidemic prevention and control system, and reducing the occupational risk of frontline medical workers is also an important guarantee to effectively control the spread of the epidemic and maintain public health safety (1, 10). At the same time, to prevent and control COVID-19 infections in medical institutions, cultivating a proper personal protection attitude is the primary prerequisite for responding to infectious disease events.

The knowledge-attitude-practice (KAP) concept is a structured survey method that has been widely used in the fields of sociology and psychology, and in recent years it has been increasingly used in the field of medicine (11, 12). A KAP survey allows the understanding of the current status of knowledge (K), attitude (A), and practice (P) of a population and explores the potential problems in the current status to provide a basis for the further optimization of health education and management strategies of the population. Still, KAP surveys have shortcomings. Indeed, they represent the KAP of a specific population from a specific location at a precise point in time, leading to poor generalizability. In addition, the questionnaire is usually designed by local investigators according to their experience and local guidelines and regulations, lacking comparability with other KAP surveys. In addition, information, selection, and social acceptability biases can be involved (12, 13). Still, the KAP theory has been widely used in the study of the impacts of COVID-19 on social life, professional work, and society. A study examined the impact of the information sources on the COVID-19 KAP of university students, showing that only a few relied on medical workers as an information sources (14). Another study examined the relationship among COVID-19, anxiety, and KAP (15).

The radiology department plays an important role in the diagnosis and management of patients with COVID-19, and the implementation of standardized X-ray and CT examination techniques is an effective guarantee for the screening, early diagnosis, and efficacy evaluation of patients with COVID-19. Several studies examined the KAP of medical workers at the forefront of the fight against COVID-19, e.g., nurses and workers from the emergency, respiratory, and cardiology departments (16–18). Still, few studies specifically examined the KAP of radiology medical workers. These medical workers not only helped in the COVID-19 pandemic but are also central to the normal activities of the hospitals.

Therefore, this study aimed to explore the KAP on the prevention and diagnosis of COVID-19 among medical workers in the radiology department.

## Materials and methods

### Study design and participants

**Figure 1:**
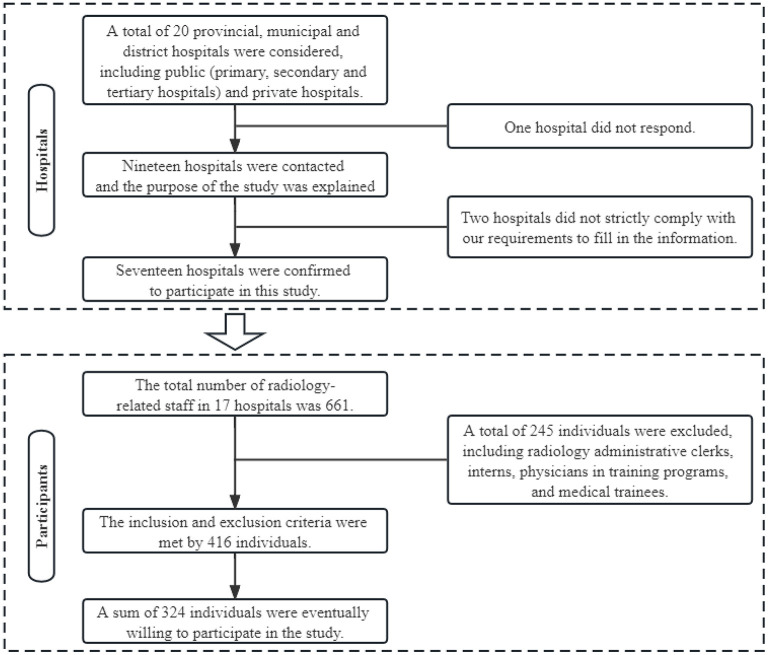
The flow diagram of the study.

This study was reported using the STROBE guidelines for cross-sectional studies (19). A total of 20 provincial, municipal, and district hospitals were considered for initial contact, including public (primary, secondary, and tertiary hospitals) and private hospitals. Responses were obtained from 19 of them. Since two hospitals did not strictly comply with our requirements for filling in the information, 17 participating hospitals were finally included (Figure 1). This multicenter website-based cross-sectional study was conducted among medical workers in the radiology department of 17 hospitals (Supplementary Table S1) between March and June 2022. The inclusion criteria were (1) radiology medical workers, including doctors, technicians, and nurses, and (2) work scope, including CT machine room, operation room, diagnostic room, and injection room. The exclusion criteria were (1) students, interns, and postgraduates or (2) retired personnel before 2019, as they did not participate in the prevention and control of the COVID-19 epidemic. The study was approved by the Institutional Review Board of The Affiliated Hospital of BeiHua University (2022-51). Electronic informed consent was obtained, and all participant data was anonymized. The questionnaire was distributed using the app “Questionnaire Star” (https://www.wjx.cn/) as the survey tool.

### Procedures

This study used a self-designed questionnaire based on the Expert Consensus on Imaging and Diagnostic Specifications for Novel Coronavirus Pneumonia (Version 1), Work Plan for Imaging and Infection Prevention and Control of Infected Pneumonia, and Medical Institutions Environmental Surface Cleaning and Disinfection Management Norms for Institutions. The questionnaire consisted of four parts: (1) the participants' demographic and socioeconomic characteristics (including education status, gender, age, practitioner type, hospital type and professional title); (2) participants' knowledge related to COVID-19 (23 questions containing possible correct answer in both COVID-19 related knowledge and knowledge of imaging diagnosis of COVID-19; one point awarded if the response contained all the correct answers, and 0 points otherwise, range 0–23; the knowledge score was categorized into three categories; < 60%—poor knowledge, 60%−80%—moderate knowledge, and 80%−100%—good knowledge); (3) participants' attitude toward prevention and diagnosis of COVID-19 [eight items using the 5-step Likert scale, ranging from “Extremely positive” (score 5) to “Extremely negative” (score 1), range (8–40)]; (4) participants' practice to prevention and diagnosis of COVID-19 [eight items by 5-step Likert scale ranging from “Always” (score 5) to “Never” (score 1), range (8–40)].

The first version of the questionnaire was drafted, and two rounds of expert consultations were then organized, inviting two experts to revise the content of the questionnaire in terms of necessity, feasibility, and logic. The Cronbach's α for the questionnaire was 0.875.

We selected 17 hospitals (including 10 tertiary hospitals, four secondary hospitals, one primary hospital, and two private institutions) across China by non-probabilistic convenience sampling, and a flow chart outlining our procedure can be seen in Figure 1. In order to ensure the quality of the returned questionnaires, each question of the questionnaire was set to be mandatory before submission, and the time required for participants to fill in the questionnaire had to be >3 min. In order to ensure the valid response rate of the questionnaire, we distributed the questionnaire through chiefs of staff in the radiology department. No remuneration was given to the participants.

### Statistical analysis

The sample size was calculated as five times the number of items in the questionnaire (20) plus 20% to account for the invalid questionnaires.

Statistical analysis was performed using SPSS 26.0 (IBM, Armonk, NY, USA) and Stata 17.0 (Stata Corp-College Station-TX-USA). Continuous data were expressed as means ± standard deviation (SD); Student's *t*-test was used for comparison between two groups, and one-way ANOVA with Tukey's *post hoc* test was used for continuous variables with three or more groups. The categorical data were presented as *n* (%) and compared with the chi-square test. Subgroup analyses were performed by practitioner type and hospital type. The practitioner and hospital types were grouped into two categories and treated as dichotomous categorical variables. Statistical analyses were conducted to investigate the differences in knowledge, attitude, and practice dimensions. Univariable and multivariable logistic regression analyses, including binary and linear logistic regression, were used to analyze the effects of basic information, knowledge, and attitude scores on practice. In addition to knowledge and attitude scores, the variables with *P*-values < 0.05 in the univariable logistic regression analyses were included in the multivariable logistic regression model to control confounding. For binary outcomes, the median was used as the cut-off value for the practice score. The KAP model assumes that better knowledge leads to more positive attitudes, which in turn leads to better practices or behaviors (12, 21, 22). Therefore, we proposed three hypotheses: (1) Good knowledge leads to a positive attitude; (2) Good knowledge brings about better practice; (3) Good knowledge causes better practices by prompting more positive attitudes. Path analysis was used to test the hypothetical model, adjusting for two additional variables. A two-sided *P*-value < 0.05 was considered statistically significant.

## Results

### Characteristics of the participants

A total of 661 radiology-related staff in the 17 participating hospitals. After excluding 245 radiology administrative clerks, interns, residents, and medical trainees, 416 individuals met the inclusion criteria, and a total of 324 individuals were ultimately willing to participate in this study (Figure 1). A total of 324 medical workers were recruited in this study, including 110 (34.0%) men. Most of them (52.2%) had an undergraduate degree. Two-fifth (40.1%) of the participants were younger than 30 years. About half (53.7%) of the participants were physicians. Most respondents (62.0%) were from tertiary hospitals. Only 13 (4.0%) respondents had a senior title. For the attitude score, significant differences were observed in age (*P* < 0.001), practitioner type (*P* = 0.003), hospital type (*P* < 0.001), and professional title (*P* < 0.001). There were significant associations of age (*P* = 0.022), practitioner type (*P* = 0.046), and professional title (*P* = 0.019) with the practice score (Table 1).

**Table 1:** Basic information of participants and KAP score.

	***n* (%)**	**Knowledge score**			**Attitude score**			**Practice score**		
		**Mean**	**SD**	***P*** **-value**	**Mean**	**SD**	***P*** **-value**	**Mean**	**SD**	***P*** **-value**
**Score**		**15.3**	**3.4**		**31.1**	**5.6**		**35.1**	**4.4**	
**Education**										
Vocational education	71 (21.9)	15.3	3.5	0.067	31.8	5.9	0.459	35.9	3.5	0.178
Undergraduate degree	169 (52.2)	14.9	3.5		31.0	5.6		34.9	4.8	
≥Postgraduate degree	84 (25.9)	16.0	3.1		30.8	5.5		34.8	4.4	
**Gender**										
Male	110 (34.0)	15.7	3.8	0.099	31.0	5.9	0.777	34.5	4.8	0.079
Female	214 (66.1)	15.1	3.2		31.2	5.5		25.4	4.2	
**Age (years)**										
< 30	130 (40.1)	15.1	3.7	0.094	29.7	5.9	< 0.001	34.4	5.2	0.022
31–40	102 (31.5)	16.0	2.9		30.7	4.8		34.9	3.8	
41–50	50 (15.4)	14.7	3.2		33.2	5.2		36.0	3.8	
>50	42 (13.0)	15.0	4.0		34.2	5.4		36.5	3.2	
**Practitioner type**										
Physician	174 (53.7)	15.0	3.7	0.001	30.4	5.8	0.003	34.6	4.6	0.046
Technician	92 (28.4)	16.4	2.9		31.0	5.3		35.3	3.5	
Nurse	58 (17.9)	14.5	3.0		33.3	5.0		36.2	5.0	
**Hospital type**										
Primary hospital	13 (4.0)	11.4	4.4	< 0.001	27.2	6.7	< 0.001	34.3	3.8	0.175
Secondary hospital	67 (20.7)	14.3	2.9		34.0	4.8		36.1	5.0	
Tertiary hospital	201 (62.0)	15.8	3.3		30.7	5.3		34.8	4.0	
Private institution	43 (13.3)	15.7	3.3		29.5	6.3		34.9	5.2	
**Professional title**										
No professional title	34 (10.5)	14.7	4.1	0.702	27.9	4.9	< 0.001	34.0	3.7	0.019
Junior title	128 (39.5)	15.5	3.2		30.5	5.6		34.8	4.7	
Intermediary title	94 (29.0)	15.4	3.1		31.3	4.9		35.2	4.1	
Vice-senior title	55 (17.0)	15.0	3.7		34.2	5.5		36.6	3.2	
Senior title	13 (4.0)	15.3	4.4		31.2	7.9		33.2	8.1	

The bold values are the Mean and SD of the total score for Knowledge, Attitude, and Practice, respectively.

### Knowledge dimension

**Figure 2:**
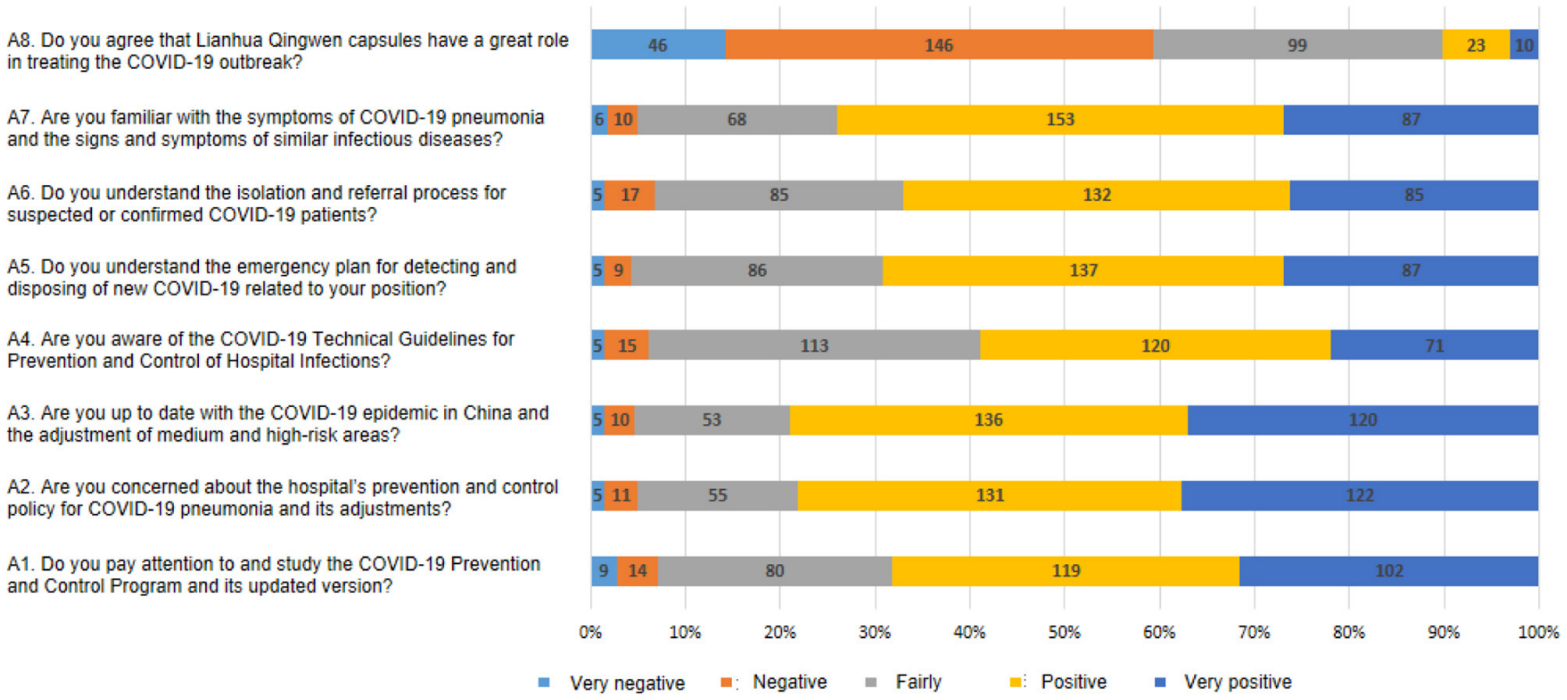
Summary of questions and responses of attitude.

**Figure 3:**
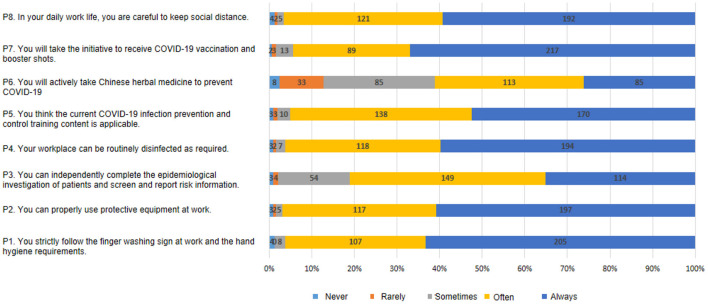
Summary of questions and responses to practice.

The mean overall knowledge score was 15.3 ± 3.4 (out of 23). There were significant knowledge score differences among participants with different practitioner types (*P* = 0.001) and hospital types (*P* < 0.001; Table 1). “The timing of hand washing/sanitization” had the highest percentage of correct answers (95.4%), while “Disinfectant capable of inactivating COVID-19” had the lowest correct rates (26.5%; Table 2). The mean attitude score was 31.1 ± 5.6 (range 8–40). The participants responded positively to all statements except “Prevention of COVID-19 by Lianhua Qingwen capsules” (Figure 2). The mean practice score was 35.1 ± 4.4 (range 8–40). The participants negatively reacted to the statement “Taking Traditional Chinese Medicine for prevention of COVID-19” (Figure 3).

**Table 2:** Responses to the questionnaire on knowledge.

**Statements**	**Responses**, ***n*** **(%)**	
	**True**	**False**
**COVID-19 related knowledge**		
K1. COVID-19 cannot be inactivated by chlorhexidine	86 (26.5)	238 (73.5)
K2. The timing of hand washing/sanitization	309 (95.4)	15 (4.6)
K3. The main route of transmission of COVID-19	143 (44.1)	181 (55.9)
K4. In order to control the spread of COVID-19 infection, medical personnel should detect and report COVID-19 infection early for early diagnosis and early isolation and treatment	268 (82.7)	56 (17.3)
K5. In China, pneumonia with COVID-19 infection is a category B infectious disease	190 (58.6)	134 (41.4)
K6. The reporting time for COVID-19 pneumonia is 2 h	188 (58.0)	136 (42.0)
K7. Patients with COVID-19 pneumonia are concentrated in the age group of 40–60 years	133 (41.1)	191 (59.0)
K8. The incubation period for COVID-19	237 (73.2)	87 (26.9)
K9. Level of protection for medical staff in fever clinics and infectious disease units	129 (39.8)	195 (60.2)
K10. Patients classified as severe or critical could be treated with convalescent plasma	214 (66.1)	110 (34.0)
K11. IgM is the first antibody to appear after infection	216 (66.7)	108 (33.3)
K12. If vaccination cannot be completed with the same vaccine from the same manufacturer, vaccination can be completed with vaccine products from other manufacturers of the same type	267 (82.4)	57 (17.6)
K13. Two drugs are approved by the National Medical Products Administration (NMPA) in China	217 (67.0)	107 (33.0)
K14. The method of seven-step hand washing	185 (57.1)	139 (42.9)
K15. The use of surgical masks	240 (74.1)	84 (25.9)
K16. The way of undressing protective clothing for medical use	202 (62.4)	122 (37.7)
K17. The way of changing disposable outer gloves	278 (85.8)	46 (14.2)
K18. The way of wearing protective clothing	293 (90.4)	31 (9.6)
**Knowledge of imaging diagnosis of COVID-19**		
K19. Chest CT findings of COVID-19 pneumonia	170 (52.5)	154 (47.5)
K20. The diagnostic value of radiologic findings	278 (85.8)	46 (14.2)
K21. Radiological diagnosis is the method of confirming the diagnosis of pneumonia in COVID-19 infections	202 (62.4)	122 (37.7)
K22. Radiological manifestations of clinical cure	259 (79.9)	65 (20.1)
K23. Common CT manifestations	244 (75.3)	80 (24.7)

The results of the subgroup analyses of practitioner type showed significant heterogeneity of total score of knowledge (*P* < 0.001), COVID-19 related knowledge (Knowledge 1–18; *P* = 0.003), and knowledge of imaging diagnosis of COVID-19 (Knowledge 19–23; *P* < 0.001) in the technicians compared with physician and nurse (Table 3). The results of the subgroup analyses of hospital type revealed significantly higher levels of knowledge in medical staff of tertiary hospitals compared with those in non-tertiary ones (Table 4).

**Table 3:** Subgroup analysis by different practitioner types.

**Variables**	**Participants**		***P*-value**
	**Technician (*****n*** = **92)**	**Physician and nurse (*****n*** = **232)**	
**Knowledge**			
Total score	16.4 ± 2.9	14.8 ± 3.5	< 0.001
COVID-19-related knowledge (knowledge 1–18)	12.5 ± 2.5	11.4 ± 2.9	0.003
Knowledge of imaging diagnosis of COVID-19 (knowledge 19–23)	3.9 ± 1.0	3.4 ± 1.2	< 0.001
**Attitude**			
Total score	31.0 ± 5.3	31.2 ± 5.8	0.869
Prevention and control policies (attitude 1–5)	19.7 ± 3.7	19.7 ± 4.1	0.890
Individual prevention (attitude 6–8)	11.4 ± 1.9	11.4 ± 4.4	0.853
Lianhua Qingwen capsules have an established role in COVID-19 (attitude 8)	3.6 ± 0.8	3.6 ± 0.9	0.752
**Practice**			
Total score	35.3 ± 3.5	35.0 ± 4.8	0.610
Protective behaviors in work (practice 1–5)	22.4 ± 2.3	22.2 ± 3.2	0.590
Protective behaviors in life (practice 6–8)	12.9 ± 1.7	12.8 ± 2.0	0.720
Taking traditional Chinese medicine for prevention (practice 6)	3.8 ± 1.1	3.7 ± 1.1	0.311

**Table 4:** Subgroup analysis by different hospital types.

**Variables**	**Participants**		***P*-value**
	**Tertiary hospital (*****n*** = **201)**	**Non-tertiary hospital (*****n*** = **123)**	
**Knowledge**			
Total score	15.8 ± 3.3	14.5 ± 3.4	0.001
COVID-19-related knowledge (knowledge 1–18)	12.0 ± 2.8	11.3 ± 2.9	0.036
Knowledge of imaging diagnosis of COVID-19 (knowledge 19–23)	3.8 ± 1.1	3.2 ± 1.3	< 0.001
**Attitude**			
Total Score	30.7 ± 5.3	31.7 ± 6.1	0.117
Prevention and control policies (attitude 1–5)	19.5 ± 3.7	20.1 ± 4.4	0.233
Focus on the prevention and control policies of the hospital (attitude 2)	4.0 ± 0.9	4.2 ± 1.0	0.094
Individual prevention (attitude 6–8)	11.2 ± 2.0	11.7 ± 2.1	0.056
Lianhua Qingwen capsules have an established role in COVID-19 (attitude 8)	3.5 ± 0.9	3.7 ± 0.9	0.083
**Practice**			
Total score	34.8 ± 4.0	35.5 ± 5.0	0.180
Protective behaviors in work (practice 1–5)	22.1 ± 2.8	22.4 ± 3.2	0.383
Cleaning and disinfection were routinely performed (practice 4)	4.5 ± 0.6	4.6 ± 0.7	0.498
Current training content was applicable (practice 5)	4.4 ± 0.7	4.5 ± 0.7	0.513
Protective behaviors in life (practice 6–8)	12.7 ± 1.8	13.1 ± 2.1	0.074
Taking traditional Chinese medicine for prevention (practice 6)	3.6 ± 1.0	3.9 ± 1.1	0.075

### Attitude and practice dimensions

For the attitude score, significant differences were observed in age (*P* < 0.001), practitioner type (*P* = 0.003), hospital type (*P* < 0.001), and professional title (*P* < 0.001). There were significant associations of age (*P* = 0.022), practitioner type (*P* = 0.046), and professional title (*P* = 0.019) with the practice score (Table 1). As shown in Table 5, positive attitudes (OR = 1.235, 95% CI: 1.162–1.311, *P* < 0.001) and ages 41–50 years were independently associated with higher practice scores. Those who had better practices were more likely to be older (OR = 2.603, 95% CI: 1.242–5.452, *P* = 0.011), nurses (OR = 2.274, 95% CI: 1.210–4.272, *P* = 0.011) and with junior/intermediary/vice-senior title (OR = 2.326, 95% CI: 1.030–5.255, *P* = 0.042; OR = 2.847, 95% CI: 1.226–6.606, *P* = 0.015; OR = 4.547, 95% CI: 1.806–11.452, *P* = 0.001, respectively). Linear logistic regression provided the same results (Supplementary Table S2).

**Table 5:** Univariable and multivariable logistic regression.

**Factors**	**Univariable logistic regression**		**Multivariable logistic regression**	
	**OR (95%CI)**	***P*-value**	**OR (95%CI)**	***P*-value**
Knowledge score	1.051 (0.985, 1.121)	0.131	0.968 (0.888, 1.054)	0.453
Attitude score	1.231 (1.167, 1.300)	< 0.001	1.235 (1.162, 1.311)	< 0.001
**Education status**				
Vocational education	Ref.			
Undergraduate degree	0.784 (0.449, 1.370)	0.393		
≥Postgraduate degree	0.775 (0.411, 1.462)	0.431		
**Gender**				
Male	Ref.			
Female	1.160 (0.732, 1.838)	0.527		
**Age (years)**				
< 30	Ref.			
31–40	1.122 (0.667, 1.885)	0.664	0.546 (0.233, 1.280)	0.164
41–50	1.485 (0.770, 2.862)	0.238	0.279 (0.080, 0.977)	0.046
>50	2.603 (1.242, 5.452)	0.011	0.515 (0.142, 1.871)	0.313
**Practitioner type**				
Physician	Ref.			
Technician	0.823 (0.495, 1.366)	0.451	0.714 (0.383, 1.332)	0.290
Nurse	2.274 (1.210, 4.272)	0.011	1.778 (0.818, 3.864)	0.146
**Hospital type**				
Primary hospital	Ref.			
Secondary hospital	3.061 (0.898, 10.429)	0.074		
Tertiary hospital	1.553 (0.491, 4.910)	0.454		
Private institution	1.267 (0.356, 4.507)	0.715		
**Professional title**				
No professional title	Ref.			
Junior title	2.326 (1.030, 5.255)	0.042	1.913 (0.757, 4.835)	0.170
Intermediary title	2.847 (1.226, 6.606)	0.015	2.952 (0.859, 10.143)	0.086
Vice-senior title	4.547 (1.806, 11.452)	0.001	3.393 (0.795, 14.488)	0.099
Senior title	2.800 (0.751, 10.445)	0.125	2.591 (0.405, 16.591)	0.315

### Path analysis

**Figure 4:**
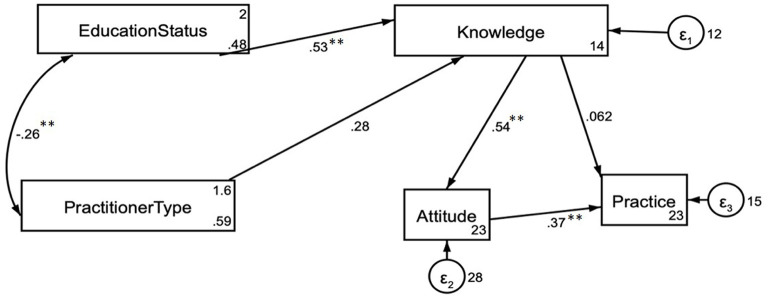
Results of structural equation modeling for the association among Knowledge, Attitude, and Practice. **P < 0.001.

As hypothesized, knowledge is positively correlated with attitude (β = 0.54, *P* < 0.001), and attitude is positively correlated with practice (β = 0.37, *P* < 0.001). Attitudes significantly mediated the association between knowledge and practice (β = 0.119, *P* < 0.001). In addition, education status tends to be significantly correlated with practitioner type (β = −0.26, *P* < 0.001), and education status directly affects knowledge (β = 0.53, *P* < 0.001; Figure 4).

## Discussion

In this study, radiology medical workers had moderate knowledge but better attitudes and practices in preventing and diagnosing COVID-19. Attitudes were found to be positively associated with better practices of prevention and diagnosis of COVID-19, and attitudes significantly mediated the association between knowledge and practice. These results indicated the need for more educational campaigns on COVID-19 among radiology medical workers and might help design and improve education strategies to improve the COVID-19 KAP in these medical workers.

These results were in agreement with a study from Singapore (23), while studies from Yemen (24) and India (25) showed poor KAP among radiology workers. Most participants were female due to the large proportion of female nurses, technicians, and physicians (23, 26). The study group mainly comprised medical workers >30 years of age and holding at least an intermediary title. The literature previously focused on young and inexperienced workers, and although the KAP gaps in this population must indeed be characterized to improve them (27), it is important to include workers of all levels to represent the clinical reality.

The results suggested that the radiology medical workers showed moderate knowledge about the prevention and diagnosis of COVID-19, but attitudes and practices were higher. Indeed, knowledge and attitudes about a specific disease will improve an individual's coping strategy toward it, resulting in better practices (28), as previously shown during a flu pandemic (29) and as shown in the present multivariable analysis. Hence, education and training about diseases could improve the practices related to that disease, as well as for other diseases in case of infectious ones, and it has been suggested that training should be compulsory during pandemics (30). Studies showed that the fear of COVID-19 vaccine side effects and improper knowledge of the vaccination benefits were determinants of hesitancy toward a COVID-19 vaccine booster dose (31, 32), highlighting the need to improve the knowledge on all aspects of COVID-19. In usual times, because most imaging machines cannot be moved, the radiology department of a hospital receives patients from all departments, including the infectious disease department and isolation wards. Therefore, radiology medical workers are already familiar with the procedures to be taken in the presence of infectious diseases (33). In the presence of an infectious disease without a vaccine or cure, as was the case with COVID-19 at the beginning of the pandemic, knowledge about the disease is vital (34). Guidelines are an important bridge between evidence and clinical practice. The body of evidence supporting the recommendations of guidelines needs to be derived from the most recent clinical research data, and therefore guidelines are continuously updated, which may result in the current version of the guidelines not necessarily covering the most current views, resulting in incorrect answers to some of the questions in the knowledge dimension.

A characteristic of a pandemic is a general feeling of panic that can give rise to misinformation in the media (35). It is, therefore, essential that governments and stakeholders implement appropriate education and training to curb misinformation from the start and ensure the best knowledge possible (36). During the avian flu pandemic, most medical workers feared the high risk of exposure and falling ill (37). During SARS in Canada, medical workers were worried about fulfilling their professional duties and responsibilities and the risk of infecting their families (38). Being torn between duty and fear of the disease for oneself and loved ones leads to anxiety (39). As the frontline of the COVID-19 outbreak response, healthcare workers are exposed to a huge risk of infection. Therefore, the psychological impact on medical care workers should be considered in future outbreaks.

This study had limitations. First, the recruitment method was not random. Second, KAP surveys are a picture of a specific situation in a specific population at a specific time point (12, 13). Therefore, the results of the present study represent only the situation of radiology medical workers in one area in China March–June 2022. Still, the data can provide a comparator for the evaluation of the effect of training in the future. This study relied on a self-reported questionnaire. A limitation of the KAP survey is the social acceptability bias, in which the participants can be tempted to answer what is considered socially or professionally acceptable instead of what they are actually doing (12, 13). Despite validation steps, there are difficulties in formulating knowledge-related questions. The content of the questionnaire was limited, and the questions developed for the knowledge dimension were difficult and might not exactly represent the knowledge about COVID-19, resulting in a low score overall. In addition, the questions are developed by the local investigators and can be influenced by local guidelines, practices, and habits. Hence, there are information and response biases. The generalizability of KAP surveys is poor unless they are performed nationwide, which was not the case here, but such a large-scale study could be planned in the future. Participation was voluntary, and there is a possibility of lower participation from less-experienced workers because of less free time available for responding or a lack of interest. In addition, anxiety and depression were not evaluated.

In conclusion, radiology medical workers showed moderate knowledge but better attitudes and practices of prevention and diagnosis of COVID-19. Attitudes were found to be positively associated with better practices of prevention and diagnosis of COVID-19, and attitudes significantly mediated the association between knowledge and practice. The results of the present study could be used to improve the education and training programs for medical workers.

## Data availability statement

The original contributions presented in the study are included in the article/Supplementary material, further inquiries can be directed to the corresponding author.

## Ethics statement

The studies involving human participants were reviewed and approved by the Institutional Review Board of The Affiliated Hospital of BeiHua University (2022-51). Written informed consent for participation was not required for this study in accordance with the national legislation and the institutional requirements.

## Author contributions

QS, YL, and CY contributed to the conception and design of the study. QS, ZZ, QW, JZ, and PJ were extracted the data. QS, CY, and QW were analysis the study. QS led and wrote the first draft of the manuscript, with the contribution of YL. All authors contributed to the manuscript revision, read, and approved the submitted version.
