# Supplementary material for: Knowledge, attitude, and practices on COVID-19 prevention and diagnosis among medical workers in the radiology department: A multicenter cross-sectional study in China
Source: Front Public Health. 2023 Mar 2;11:1110893. doi: 10.3389/fpubh.2023.1110893 (PMC10018012; doi:10.3389/fpubh.2023.1110893)
Supplement: Supplementary file 1 [file Table_1.pdf]

Supplementary Table S1. Participating centers

Hospital type	Hospital designation
Tertiary hospitals	Longgang District Central Hospital of Shenzhen
	Shenzhen People’s Hospital
	Peking University Shenzhen Hospital
	Guilin People’s Hospital
	Affiliated Hospital of Beihua University
	Chinese and Mongolion Hospital of Zhalantun
	Jilin Province People’s Hospital
	Beijing Aerospace General Hospital
	Peoples Hospital of Zhongshan
	Ansteel Group Hospital
Secondary hospitals	Shenzhen Second People’s Hospital
	Liaoyuan Maternity and Infant Hospital
	Second Hospital of Changchun
	Huadian People’s Hospital
Primary hospital	Shenzhen Longgang District Seventh People’s Hospital
Private institutions	Medical Imaging Center of Meridian Health Shenzhen Branch
	Medical Imaging Center of Meridian Health Guangzhou Branch

Supplementary Table S2. Univariable and multivariable linear regression

Factors	Univariable linear regression		Multivariable linear regression	
	B (95%CI)	P	B (95%CI)	P
Knowledge score	0.012 (-0.004, 0.028)	0.130	-0.007 (-0.023, 0.009)	0.370
Attitude score	0.041 (0.033, 0.050)	<0.001	0.042 (0.032, 0.052)	<0.001
**Education status**				
Vocational education	Ref.			
Undergraduate degree	-0.060 (-0.200, 0.079)	0.395		
≥ Postgraduate degree	-0.063 (-0.222, 0.096)	0.434		
**Gender**				
Male	Ref.			
Female	0.037 (-0.079, 0.153)	0.528		
**Age (years)**				
<30	Ref.			
31-40	0.029 (-0.101, 0.158)	0.663	-0.126 (-0.289, 0.037)	0.130
41-50	0.098 (-0.064, 0.261)	0.235	-0.251 (-0.483, -0.020)	0.034
>50	0.229 (0.055, 0.403)	0.010	-0.137 (-0.372, 0.098)	0.253
**Practitioner type**				
Physician	Ref.			
Technician	-0.049 (-0.174, 0.077)	0.447	-0.068 (-0.189, 0.053)	0.269
Nurse	0.195 (0.048, 0.343)	0.010	0.111 (-0.034, 0.257)	0.133
**Hospital type**				
Primary hospital	Ref.			
Secondary hospital	0.272 (-0.024, 0.568)	0.072		
Tertiary hospital	0.108 (-0.172, 0.388)	0.448		
Private institution	0.057 (-0.252, 0.367)	0.716		
**Professional title**				
No professional title	Ref.			
Junior title	0.198 (0.010, 0.386)	0.039	0.134 (-0.041, 0.309)	0.133
Intermediary title	0.248 (0.054, 0.443)	0.013	0.222 (-0.011, 0.455)	0.062
Vice-senior title	0.360 (0.148, 0.573)	0.001	0.249 (-0.020, 0.519)	0.070
Senior title	0.244 (-0.073, 0.562)	0.131	0.217 (-0.122, 0.557)	0.208
